# What is the right temperature to cool post-cardiac arrest patients?

**DOI:** 10.1186/s13054-015-1134-z

**Published:** 2015-11-18

**Authors:** Premkumar Nattanmai Chandrasekaran, Cameron Dezfulian, Kees H. Polderman

**Affiliations:** Department of Critical Care Medicine, University of Pittsburgh, Scaife Hall, 3550 Terrace Street, Pittsburgh, PA 15261 USA

## Abstract

**Citation:**

Niklas Nielsen, Wetterslev J, Cronberg T, Erlinge D, Gasche Y, Hassager C, Horn J, Hovdenes J, Kjaergaard J, Kuiper M, Pellis T, Stammet P, Wanscher M, Wise MP, Åneman A, Al-Subaie N, Boesgaard S, Bro-Jeppesen J, Brunetti I, Bugge JF, Hingston CD, Juffermans NP, Koopmans M, Køber L, Langørgen J, Lilja G, Møller JE, Rundgren M, Rylander C, Smid O, Werer C, Winkel P, Friberg H. Targeted temperature management at 33 °C versus 36 °C after cardiac arrest. N Engl J Med. 2013;369:2197–206. doi:10.1056/NEJMoa1310519. Epub 2013 Nov 17. Pub Med PMID: 20089970.

**Background:**

Brain ischemia and reperfusion injury leading to tissue degeneration and loss of neurological function following return of spontaneous circulation after cardiac arrest (CA) is a well-known entity. Two landmark trials in 2002 showed improved survival and neurological outcome of comatose survivors of out-of-hospital cardiac arrest (OHCA) of presumed cardiac origin when the patients were subjected to therapeutic hypothermia of 32 to 34 °C for 12 to 24 hours. However, the optimal target temperature for these cohorts is yet to be established and also it is not clear whether strict fever management and maintaining near normal body temperature are alone sufficient to improve the outcome.

**Methods:**

*Objective:* The objective is to determine whether a hypothermic goal of a near-normal body temperature of 36 °C reduces all-cause mortality compared with a moderate hypothermia of 33 °C for the unconscious survivors of OHCA of presumed cardiac origin when subjected randomly to these different targeted temperatures.

*Design:* A multicenter, international, open label, randomized controlled trial.

*Setting:* Thirty-six ICUs in Europe and Australia participated in this study.

*Participants:* Unconscious adults (older than 18 years of age) who survived (Glasgow coma scale less than 8) OHCA due to presumed cardiac origin with subsequent persistent return of spontaneous circulation (more than 20 minutes without chest compressions).

*Intervention:* The above participant cohorts were randomized to targeted body temperature of either 33 °C or 36 °C for 36 hours after the CA with gradual rewarming of both groups to 37 °C (hourly increments of 0.5 °C) after the initial 28 hours. Body temperatures in both the groups were then maintained below 37.5 °C for 72 hours after the initial 36 hours.

*Outcomes:* Primary outcome measure of all-cause mortality in both the groups at the end of the trial with the secondary outcome measure of all-cause mortality, composite neurological function as evaluated by cerebral performance category scale and modified ranking scale at the end of 180 days were studied.

**Results:**

Out of the 939 participants, all-cause mortality at the end of the trial was 50 % in the 33 °C group (225 of 466 patients) compared with 48 % in the 36 °C group (235 of 473 patients); the hazard ratio with a temperature of 33 °C was 1.06 (95 % confidence interval (CI) 0.89 to 1.28, *P* = 0.51). At the end of 180 days, 54 % of patients in the 33 °C group versus 52 % in the 36 °C group had died or had poor neurological outcome according to cerebral performance category (risk ratio 1.02, 95 % CI 0.88 to 1.16, *P* = 0.78) but the modified ranking scale at the end of 180 days was unchanged (52 %) in both groups (risk ratio 1.01, 95 % CI 0.89 to 1.14, *P* = 0.87).

**Conclusions:**

Maintaining targeted lower normothermia of 36 °C had similar outcomes compared with induced moderate hypothermia of 33 °C for unconscious survivors of OHCA of presumed cardiac cause.

## Commentary

Out-of-hospital cardiac arrest (OHCA) is a devastating and traumatic event for the patient and their family. Even if the patient survives the initial event the ischemia and the reperfusion injury lead to tissue destruction and loss of neuronal function.

One of the major breakthroughs in managing post-cardiac arrest patients is the implementation of hypothermia, which has shown promising results in improving neurological function and survival. The earliest recorded application of therapeutic cooling in medicine dates back about 5000 years; its use became widespread after publication of two randomized controlled trials (RCTs) in 2002, which demonstrated safety and efficacy of mild (32 °C to 34 °C) cooling after cardiac arrest (CA) [1, 2]. These initial trials were followed by numerous nonrandomized studies showing improved outcomes when therapeutic hypothermia was introduced [3, 4]. Based on the two RCTs and subsequent supporting evidence, the International Liaison Committee on Resuscitation and American Heart Association guidelines recommend the use of cooling to 32 to 34 °C after witnessed CA when the initial rhythm was pulseless ventricular tachycardia (VT)/ventricular fibrillation (VF) [5]. This was supported by a Cochrane analysis [6].

However, the evidence underpinning use of hypothermia in CA patients was subsequently criticized. The largest RCT had excluded more than 90 % of screened patients and had not applied temperature management in controls, where the average temperature was 37.8 °C [1]; the other RCT did successfully maintain normothermia in controls, but here randomization had been by day of the month instead of per patient [2], and differences in this (much smaller) study barely achieved statistical significance. Harmful effects of fever are well recognized [3]; based on the two RCTs, it could not be ruled out that strict fever management alone could be sufficient to prevent temperature-induced neurological damage.

This led to the initiation of a much larger multicenter RCT to compare two targeted temperature regimens (mild hypothermia of 33 °C versus near normal body temperature of 36 °C) in 939 unconscious survivors of OHCA of presumed cardiac cause after sustained return of spontaneous circulation. The intervention period of 36 hours commenced at the time of randomization and the goal was to achieve the assigned core temperature as rapidly as possible using different cooling methods and to maintain this temperature for 24 hours, followed by rewarming at a rate of 0.5 °C per hour to 37 °C and maintaining this in both groups for another 72 hours. Recommendation for continuation or withdrawal of therapy was done in a blinded fashion at 72 hours based on clinical parameters and by sensory-evoked potentials and/or electroencephalogram as indicated.

The study found that rates of the primary outcome measure (death) and secondary outcome measure (poor neurologic function) did not differ significantly between the groups. Subgroup analyses did not indicate significant benefits from either strategy. Patients presenting with more severe potential neuronal injury (for example, those with nonshockable rhythm and longer time to return of spontaneous circulation) also had no difference in outcome between interventions. The only difference in favor of the 33 °C group was in the rates of survival with excellent outcome (no neurologic residual), which was 41.6 % versus 39.4 % (*P* was not significant). The incidence of adverse events also did not differ significantly except for higher incidence of hypokalemia (*P* = 0.018) and nonsignificant trends to higher incidence of hypomagnesemia (*P* = 0.20), hypophosphatemia (*P* = 0.13) and pneumonia (*P* = 0.089) in the 33 °C group.

This study was done with fewer exclusion criteria and had a much larger study population (939 patients) than previous trials. In contrast to the previous studies it included patients with initial cardiac rhythms other than VT/VF. Another unique feature of the study is the adoption of a protocol for withdrawal of life-sustaining treatment; it followed the important concept of preventing fever by actively controlling temperature in both groups during the first 3 days after CA.

Potential criticisms include a delayed start of cooling, a prolonged time to target temperature (10 hours) and a rapid rewarming rate (from 33 °C to 36 °C in 6 hours), much faster than in previous trials. Although no comparative studies assessing optimal rewarming rates after CA have been performed, animal studies and studies in patients undergoing cardiac surgery with extracorporeal circulation strongly suggest that rapid warming can be harmful, and could negate some or all of the benefits of therapeutic cooling [7, 8]. As the trial enrolled, on average, just one patient per center per month it is conceivable that some type of pre-selection occurred, which could have influenced the results if the selection was not random [4, 9].

In spite of these potential criticisms, the fact that good outcome was achieved in just under 50 % of patients in both groups demonstrates that, in many patients, very strict fever control (36 °C) alone is sufficient to provide neuroprotection. The question still remains whether we can identify specific cohorts of CA patients who need different depth and duration of temperature management. A study by Lopez-de-Sa et al. published in 2012 found much better outcomes when patients were cooled to 32 °C compared with 34 °C [10]. The targeted temperature management trial suggests that 33 °C is not better than 36 °C in many patients. The duration of temperature management also has not been conclusively settled.

## Recommendations

CA patients should receive active temperature management after resuscitation. The targeted temperature management trial found that temperatures of 33 °C or 36 °C yielded similar outcomes, though whether this applies to all CA patients is controversial. The absence of temperature control, as was the norm before 2002, is linked to higher mortality and should be avoided.

## Abbreviations

CA: cardiac arrest
CI: confidence interval
OHCA: out-of-hospital cardiac arrest
RCT: randomized controlled trial
